# Aptamer Screening: Current Methods and Future Trend towards Non-SELEX Approach

**DOI:** 10.3390/bios14070350

**Published:** 2024-07-18

**Authors:** Zhihui Fang, Xiaorui Feng, Fan Tang, Han Jiang, Shuyuan Han, Ran Tao, Chenze Lu

**Affiliations:** 1 Key Laboratory of Specialty Agri-Products Quality and Hazard Controlling Technology of Zhejiang Province, College of Life Sciences, China Jiliang University, Hangzhou 310018, China; fangzhihui@cjlu.edu.cn (Z.F.); p22091055017@cjlu.edu.cn (X.F.); tangfan@cjlu.edu.cn (F.T.); jianghan@cjlu.edu.cn (H.J.); 2300902102@cjlu.edu.cn (S.H.); 2 Shenzhen Key Laboratory of Advanced Thin Films and Applications, College of Physics and Optoelectronic Engineering, Shenzhen University, Shenzhen 518060, China

**Keywords:** aptamer screening, RNA aptamer, XNA aptamer, non-SELEX methods, high-resolution partitioning

## Abstract

Aptamers are nucleic acid sequences that specifically bind with target molecules and are vital to applications such as biosensing, drug development, disease diagnostics, etc. The traditional selection procedure of aptamers is based on the Systematic Evolution of Ligands by an Exponential Enrichment (SELEX) process, which relies on repeating cycles of screening and amplification. With the rapid development of aptamer applications, RNA and XNA aptamers draw more attention than before. But their selection is troublesome due to the necessary reverse transcription and transcription process (RNA) or low efficiency and accuracy of enzymes for amplification (XNA). In light of this, we review the recent advances in aptamer selection methods and give an outlook on future development in a non-SELEX approach, which simplifies the procedure and reduces the experimental costs. We first provide an overview of the traditional SELEX methods mostly designed for screening DNA aptamers to introduce the common tools and methods. Then a section on the current screening methods for RNA and XNA is prepared to demonstrate the efforts put into screening these aptamers and the current difficulties. We further predict that the future trend of aptamer selection lies in non-SELEX methods that do not require nucleic acid amplification. We divide non-SELEX methods into an immobilized format and non-immobilized format and discuss how high-resolution partitioning methods could facilitate the further improvement of selection efficiency and accuracy.

## 1. Introduction

Aptamers are single-stranded oligonucleotide sequences that can specifically bind with target molecules, thus they are also referred to as “chemical antibodies”. The nature of aptamers could be traditional nucleic acids (DNA and RNA) or xeno-nucleic acids (XNA) with modifications in the backbone, sugar group or base. Compared with antibodies, aptamers have the advantages of easy modification and stable massive production, which allows more versatile applications and more consistent results [1]. Aptamers are also capable of working in bio-incompatible environments and their thermal denaturation is reversible [2]. As a result, aptamers are widely used in biosensing [3], drug delivery [4], new drug development and biomedical research [5,6].

The selection of aptamers is often accomplished by the traditional Systematic Evolution of Ligands by an Exponential Enrichment (SELEX) procedure, in which a library of random synthesized sequences is first mixed with the target, such as proteins, small molecule compounds or even ions and living cells. Various methods are applied to separate sequences with different affinities towards the target, and this process is repeated for several cycles along with elution and nucleic acid amplification processes to escalate sequences with high affinity. This approach effectively meets the requirements for DNA aptamer selection. However, it is troublesome while screening RNA or XNA aptamers since RNA requires extra transcription and reverse transcription procedures and the enzymes for DNA amplification are not always applicable to XNA [7,8,9]. However, DNA, RNA and XNA aptamers each have their own strengths. Currently, DNA aptamers are the most popular in the field of rapid detection, while RNA and XNA aptamers are rarely used due to the difficulty of screening and the high cost of synthesis. RNA aptamers are more susceptible to nuclease degradation due to the presence of the 2’ hydroxyl group, and their stability in vivo is not as good as that of DNA aptamers. Nevertheless, RNA aptamers can offer distinct advantages in specific medical contexts. For instance, RNA aptamers are able to mimic some biological functions of natural RNA, such as catalyzing reactions and regulating gene expression, and stand out in the development of RNA-based therapeutics [10,11]. Due to delicately designed modifications, XNA aptamers exhibit superior binding properties, broad chemical diversities, and reduced immunogenicities and toxicities [6]. These advantages make XNA aptamers more durable for clinical and biotechnological applications and complex environments. In addition, XNA aptamers demonstrate innovative biological activities and environmental adaptabilities, paving the way for groundbreaking opportunities in biomedical research and applications. They serve as pivotal instruments in various domains, including drug development, disease diagnostics and biosensing technologies. For these reasons, there is an urgent need for the development of novel screening methods that are more suitable for RNA and XNA aptamers. The ideal approach is to build screening systems that do not require nucleic acid amplification, so that the difficulties could be bypassed. There have been many reviews published over the last three years, but most of them focus on aptamer selection towards a specific target or application [12,13], while others emphasize a certain type of screening methods such as capillary electrophoresis [14], microfluidic chips [15], in silico methods [16] and the directed evolution of screened aptamers [17]. However, none of the recent reviews aim to organize the existing screening methods that are independent of intermediate nucleic acid amplification.

In this review, we give a brief introduction to existing aptamer screening methods followed by a detailed explanation and comparison of non-SELEX screening methods. We have categorized these methods into an immobilized format and non-immobilized format depending on whether nucleic acid aptamers are immobilized on the surface of other materials or are free-dangling in solution. Furthermore, in silico methods that are based on computer aid to rational design aptamer sequences are discussed as well. In the last section, we summarize the pros and cons of the latest research and give our perspective on the trend of future work.

## 2. SELEX-Based Selection Methods of DNA Aptamers

**Figure 1:**
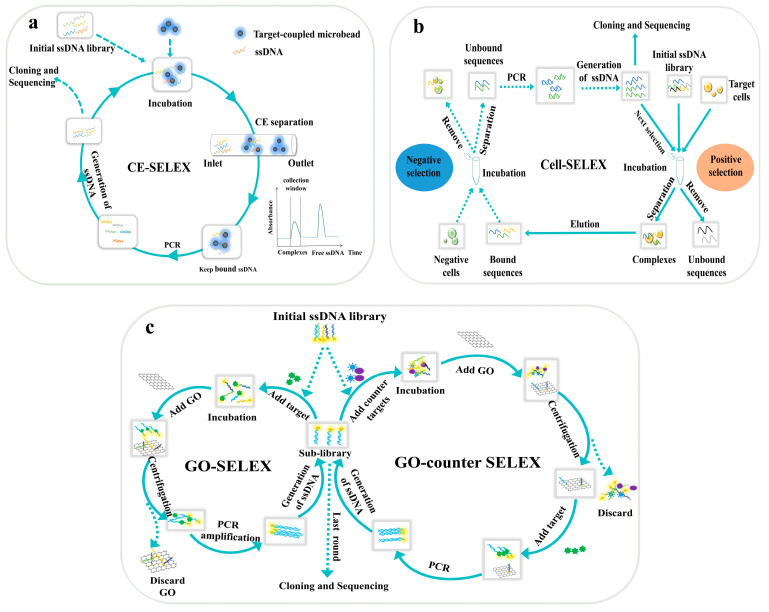
The schematic diagrams of SELEX methods in non-immobilized format. (**a**): The principle of CE-SELEX method. The method isolates substances based on electrophoretic mobility. Nucleic acid libraries are incubated with targets to form an equilibrium mixture. The components are separated due to difference in mobility in electric field [18]; (**b**): The principle of cell-SELEX method. The target molecules on the cell surface are in their native state and do not require additional purification sequences that bind with these target molecules in their natural conformation [19]; (**c**): the principle of GO-SELEX method, ssDNA already bound to targets will not be adsorbed by GO due to the change of spatial structure, which allows separation of potential aptamer sequences [20].

**Figure 2:**
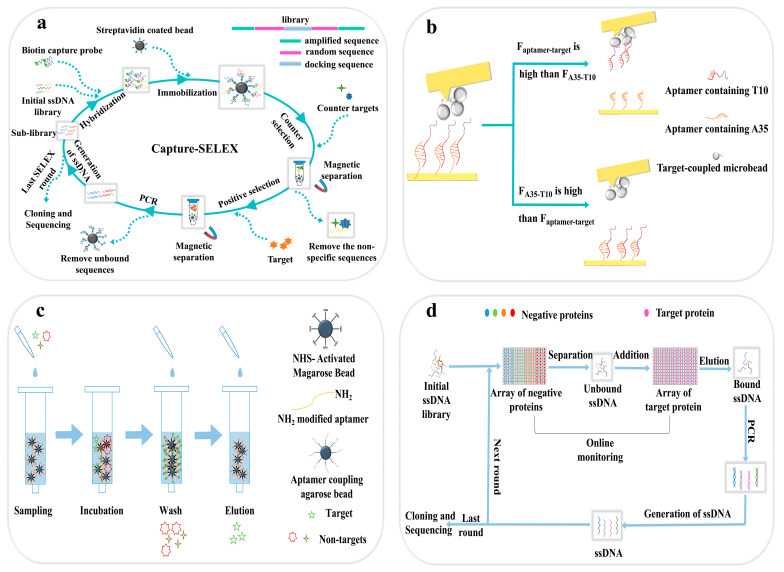
The schematic diagrams of SELEX methods in immobilized format. (**a**): The principle of capture-SELEX method. The complementary chains of the docking sequences in the library are immobilized on magnetic beads. After mixing and incubation, the conformation of the target binding sequences changes and can be released from the magnetic beads [21]; (**b**): The principle of AFM-SELEX method. A nucleic acid library is immobilized on the gold chip and the target is functionalized on the microcantilever. The aptamers is released from the gold chip when the binding affinity is high enough [22]; (**c**): The principle of affinity chromatography SELEX. The aptamers are immobilized on the surface of the magnetic beads and loaded into the affinity column. The targets interact specifically with the aptamers when samples are injected, and potential aptamers are selectively adsorbed onto the column and eluted for further analysis [23]; (**d**): Microarray-SELEX divides multiple regions on the surface of a chip, each of which can point to a target, and then the library is added to the chip and screened for specific aptamers through multiple rounds of positive and negative selection [24].

Based on the treatment of the DNA library during selection, SELEX methods could be categorized into either immobilized or non-immobilized formats. The former refers to those situations in which nucleic acid sequences are immobilized on a solid surface, including metals, plastics, membranes, gels or nanoparticles, whereas in a non-immobilized format, neither the aptamer nor the target is immobilized and both exist in a free form. Both formats are capable of screening aptamers with high specificity and affinity, but each has its pros and cons. Herein, we present the latest cases of the SELEX-based selection for DNA aptamers to offer a general view. The principles of these traditional SELEX methods are displayed in Figure 1 and Figure 2 and are sorted by the format of nucleic acid during selection. The information of the articles mentioned in this section is listed in Table 1.

### 2.1. SELEX Methods in Immobilized Format

#### 2.1.1. Capture-SELEX

The capture-SELEX method first immobilizes an initial library containing random and docked sequences, and then uses complementary strands of docked sequences labeled with biotin as the capture probes. The capture probes bind to streptavidin-labeled magnetic beads to capture the library through base pairing [50]. Zheng et al. incorporated Fluorescein Amidite (FAM) tag size preparation with Capture-SELEX and were able to screen aptamers with K_d_ as low as 25.3 nM [25]. Since Capture-SELEX immobilizes the DNA library instead of the target, it is preferable for the screening of a small target. Gu et al. obtained aptamers against salicylic acid after 17 rounds of Capture-SELEX, and the aptamer was further used for label-free fluorescent detection and displayed a low limit of detection of 2.2 μM [26]. However, Capture-SELEX suffers from a low overall success rate and is limited by a time-consuming procedure. Due to the weak base complementarity force, the immobilized library may experience a dynamic dissociation equilibrium, resulting in the dissociation of some sequences not bound to the target from the solid phase, hence leading to false positives. This phenomenon hampers the screening efficiency and increases the screening difficulty [21]. In addition, affected by the capture ability of the capture probe, not all sequences in the designed random sequence library will be captured on the solid phase, the abundance of the library may decrease and the diversity of the library cannot be well guaranteed. Therefore, it is necessary to pay attention to the design of capture-SELEX libraries, monitor the whole screening process in combination with other techniques such as quantitative real-time PCR, and improve the stringency of screening by adding a negative selection to avoid blind screening.

#### 2.1.2. Affinity Chromatography SELEX

Affinity chromatography is a high-resolution separation method widely used in analytical chemistry, which makes it an ideal technology to isolate bound aptamers and an unbound library [13]. The technique combines the principles of affinity chromatography and molecular selection and can be used to find ligands (e.g., antibodies, oligonucleotides) with a high affinity for specific molecules (e.g., proteins, nucleic acids). Commonly used sample separation techniques in affinity chromatography include immobilized metal affinity chromatography (IMAC), immunoaffinity columns (IAC) and aptamer affinity columns (AAC) [23,27]. In recent years, a variety of improved SELEX techniques have emerged, while the use of traditional affinity chromatography SELEX applications is decreasing. Affinity chromatography is reproducible but less efficient and has limited applicability. In order to remain competitive, SELEX technology based on affinity chromatography has to be continuously improved to solve practical problems [28]. In this process, exploring new strategies and discovering new means are quite challenging tasks.

#### 2.1.3. Atomic Force Microscope SELEX

An atomic force microscope (AFM) is not only used for the surface mapping of materials, but also frequently used to measure interactions between cells or molecules [51]. In general cases, a biotin-labeled single-strand DNA (ssDNA) library is connected to a streptavidin-functionalized cantilever, which taps on targets immobilized on the surface of a gold chip. The interaction between thymine and adenine could be used to eliminate sequences with a low affinity. Takenaka et al. immobilized a DNA library on the surface of 35mer adenine and 10mer thymine modified gold chips, so that aptamers with a weaker affinity would not be able to break their binding and were eliminated [52]. In some cases, an AFM is used as a tool for studying the binding between the aptamer and target. Yamamoto et al. analyzed the mechanism of target binding by preparing a DNA frame with the DNA origami technique and a fixed protein in the DNA frame so that its interaction with the aptamer could be observed with an AFM [29].

### 2.2. SELEX Methods in Non-Immobilized Format

#### 2.2.1. Capillary Electrophoresis

Capillary electrophoresis-SELEX (CE-SELEX) takes advantage of the high resolution of capillary electrophoresis to separate sequences that are bound to the target from the library, which makes it very efficient and flexible in screening mode. The most common procedure is shown in Figure 1a; the DNA library is first mixed with targets and then an electric field is applied to drive them towards the outlet. The target, single-strand DNA and their complex are separated due to the difference in mobility. Capillary electrophoresis SELEX is one of the most efficient methods for aptamer screening. It usually takes only a few rounds of screening to obtain high-affinity aptamers. For instance, Li et al. utilized CE-SELEX to screen aptamers against Tropomyosin, with good binding properties in only four rounds [18]. The introduction of magnetic beads could provide the efficient isolation and separation of aptamers and unbound DNA. Nagano et al. screened aptamers within three rounds using this method and achieved a low limit of detection (LoD) of 118 nM, which suggested a high affinity of the screened aptamers [30]. However, the drawback is that magnetic beads may hinder binding between the library and target. In a more recent case, a target-specific protein was added to the selection procedure to create a binder-enriched library. Zhao et al. established a three-step protocol that involves two rounds of sequence convergence, two rounds of positive selection and a final round of negative selection and the dissociation constant (K_d_) was measured to be 27.6 nM [31].

On the other hand, most studies consider an equilibrium state between the target and aptamer, but such an equilibrium is disturbed during partitioning. Kinetic capillary electrophoresis focuses on dynamic interactions between molecules, which offers high partitioning efficiency [53]. Le et al. drove the bound complex and unbound sequences in opposite directions; the capillary electrophoresis acted like a filter to remove unspecific sequences [32].

#### 2.2.2. Cell-SELEX

Cell-SELEX uses whole living cells as targets for selection so that no screening of surface proteins or functional groups needs to be carried out beforehand. A key point in Cell-SELEX is to maintain cells in viable conditions to avoid a potential impact on the result. Fluorescence-activated cell sorting (FACS) is developed to differentiate between live and dead cells based on fluorescent signals and light scattering properties. Yoshikawa et al. demonstrated the successful screening of highly specific aptamers against various glycosylated forms of proteins using FACS [33]. The same as CE-SELEX, introducing a competing ligand leads to aptamers with a higher affinity and specificity by minimizing the impact of non-specific sequences [13]. But this approach is only applicable when a secondary ligand with high affinity against the target exists. Due to the complexity of the cell surface and cell viability conditions, the whole screening normally takes multiple rounds and many days or even weeks to finish [54]. Microfluidic chips were combined with Cell-SELEX to accelerate and improve automation. Tsai et al. screened aptamers against cell membranes of ovarian cancer tissues with the help of the on-chip method and successfully reduced the whole selection time to 15 h [34].

The cell condition is vital during selection, not just in their basic survival, but also related to the morphology and growing conditions. Cells tend to display flat structures in traditional two-dimensional (2D) culture models, which affects the screening results through the cell morphology. Nelissen et al. used three-dimensional (3D) spheroids of breast cells to generate aptamers for breast cancer treatment, which is the first case of introducing spheroids to aptamer selection [35]. The result was confirmed with therapeutic tests and proved that the positive selection with malignant spheroids and negative selection with non-malignant spheroids were both effective.

#### 2.2.3. Graphene Oxide-SELEX

Graphene oxide-SELEX (GO-SELEX) is based on the adsorption of free ssDNA on the GO surface due to a π–π interaction, which could help separate the unbound ssDNA and potential aptamers bound with targets. Screening aptamers for small molecules in vitro is challenging due to the limited functional groups on the surface of small molecules that can be utilized for coupling, and even fewer binding sites are exposed after binding to solid-phase materials [55]. Most screening methods that require immobilizing targets are not suitable for small molecules. As no target immobilization is required, GO-SELEX holds great promise for screening aptamers against biomolecules and small molecules. The optimization of the amount of GO is an essential parameter of GO-SELEX, since it directly affects the partitioning efficiency. Yang et al. explored the influence of the ratio between the GO mass and ssDNA and noticed that the recovery rate decreased with this ratio [20]. Thanks to the fluorescence-quenching effect of GO, the fluorescent label could be added to optimize the GO/ssDNA mass ratio. Hedayati et al. utilized GO as a quencher of a fluorescent assay during SELEX, and reported the successful selection of high-affinity aptamers (K_d_ = 178.4 nM) [36]. GO is an ideal material for the preparation of biosensors due to its high chemical stability and good biocompatibility. However, the limited solubility of graphene oxide, which may lead to unsatisfactory separation, and the GO molecule needs to be modified to improve its solubility [13]. In addition, target-bound ssDNA can be separated from unbound ssDNA by centrifugation during GO-SELEX. While performing the separation, a certain amount of graphene oxide may enter the supernatant, and the unbound ssDNA present on the surface of the graphene oxide contaminates the target-bound ssDNA pool, which may require more SELEX cycles to obtain the target-specific aptamer if the pool is amplified during the SELEX amplification step [56]. This is a little shortcoming of GO-SELEX technology.

## 3. SELEX-Based Selection Methods of RNA and XNA Aptamers

### 3.1. RNA Aptamer Selection

The typical protocol for screening RNA aptamers introduces an additional reverse-transcription step before the amplification step, whereas the amplified library has to undertake another transcription step for the next round of screening. Due to the necessity of these steps, RNA aptamer selections seldom apply chemical modifications to the library, limiting the choices of selection strategies. Other than the extension of the whole screening process, it also increases the chance of obtaining misleading results due to mutations in these additional steps. The chance significantly increases if the whole sequence is longer than 100 nt [57]. Hence, the key in RNA aptamer selection methods is how to eliminate or minimize such results. Yeoh et al. developed a tripartite-hybrid SELEX that combined multiple separation mechanisms including a nitrocellulose filter membrane, microtiter plate and native polyacrylamide gel electrophoresis-based separation to ensure that unbound aptamers were removed more completely [37]. Vockenhuber et al. used two different kinds of magnetic beads, respectively, in odd rounds and even rounds to remove potential aptamers that were specific to magnetic beads [38].

On the other hand, RNA aptamers are more frequently used in cell therapy which comes with more sophisticated applications than target binding. Ruiz-Ciancio et al. carried out cell internalization SELEX for the screening of internalizing RNA aptamers that were designed to penetrate cell membranes [39]. Instead of washing and collecting aptamers bound to the surface of target cells, the internalization of the SELEX protocol recovers aptamers from a cell lysis solution. Although nucleic acid sequences originating from the target cells are also collected, these sequences do not take part in the amplification process, which has little impact on the final result.

An alternative approach is to establish more automatic systems to ease the experimental protocol. Microfluidic chips provide the automatic control of the fluid inside the chip and have great potential in achieving the rapid screening of RNA aptamers. Autour et al. integrated a droplet-based microfluidic chip with next-generation sequencing and built an on-chip screening system for optimizing biosensing results with RNA aptamers; the droplets that exhibited the desired fluorescent signal were sorted and collected for the next round of selection automatically [40]. Cubi et al. utilized an efficient system named microfluidic-assisted in vitro compartmentalization (μIVC) to screen droplets at the pL level [41]. With additional help from an artificial neural network (ANN), they were able to improve the performance of a model RNA aptamer by 10-fold. In this study, researchers utilized an unsupervised artificial neural network to more effectively analyze high-throughput sequencing data and streamline the information. By training the ANN algorithm with a vast amount of RNA sequence data, the researchers clustered and ordered the sequences, identifying key features within the RNA sequences which enabled the classification of RNA aptamers. This approach facilitated the rapid identification and characterization of RNA aptamers, providing a new tool and technical support for RNA structure and function research. However, based on existing studies, it is not certain that this method is necessarily applicable to the screening of all aptamers. A number of factors also need to be considered when applying the technique, such as the quality and quantity of data, the level of accuracy required, the complexity of the problem and whether the ANN is able to accurately capture the features and relationships required for aptamer screening. In practice, it may be necessary to combine other techniques and methods to achieve better screening results. But this could provide a direction for future research.

### 3.2. XNA Aptamer Selection

The term XNA refers to synthetic nucleic acids that do not appear in natural environments. XNA could carry modifications either on the sugar or phosphate of the nucleic acid backbone or the nucleobases [58]. At present, the synthesis methods of XNAs include enzymatic synthesis, chemical synthesis, biosynthesis, etc. Each of these methods has its advantages and disadvantages, and the selection of the appropriate method depends on the specific application needs and research purposes. Readers can find a detailed introduction of the synthesis and application of XNA in previous reviews by Yu et al. [59,60].

There are two conventional strategies in screening XNA aptamers, post-SELEX and modified-SELEX. The former introduces modifications after the screening of natural aptamers, whereas the latter starts from the XNA library [61]. Regardless of the difficulties in synthesizing the XNA library, the main obstacles to XNA aptamer selection are the low efficiency of the amplification enzymes and incompatibility with existing sequencing methods [62,63]. The partitioning and enrichment of potential XNA aptamer sequences still follow traditional procedures, such as CE-SELEX [42], cell-internalization SELEX [43], affinity chromatography SELEX [44], etc. Due to the structural complexity and chemical diversity of XNA, the choosing and improving of selection methods are highly dependent on real applications. In some cases, researchers even have to combine more than one SELEX method. Uemachi et al. merged CE-SELEX with cell-SELEX and developed a novel hybrid-SELEX approach [45]. In their study, CE-SELEX was first applied to pre-screen aptamers that exhibited strong binding against the surface protein of a target cell and then cell-SELEX was applied to further select the aptamers that were able to penetrate the membrane of the target cell.

Unlike RNA aptamer selection, XNA aptamer selection tends to bind the library to magnetic beads instead of targets, while using a magnetic field to improve the separation resolution. In order to determine the optimal modification to improve the aptamer performance, Siegl et al. split the library into four portions with different modifications. These portions were mixed and functionalized on magnetic beads for screening [46]. The screened sequences were amplified with PCR using C5-ethynyl-deoxyuridine triphosphate (dUTP with modification). Wang et al. screened based-modified aptamers for tumor therapy with cell-internalization SELEX. Biotin-labeled aptamers were incubated with streptavidin-coated magnetic beads for immobilization, which enabled the efficient separation of target aptamers in the cell lysis solution [43].

Since it is quite troublesome to accelerate the SELEX procedure of XNA, some measures are suggested to improve the efficiency of the binding evaluation of potential aptamers to shorten the overall time required. Yik et al. coupled potential aptamers on the gel matrix of polyacrylamide hydrogel particles instead of solidified beads, which offered a mobile environment closer to the working conditions of aptamers [47]. Dunn et al. identified α-L-threofuranosyl nucleic acid (TNA) with high affinity using a display strategy similar to protein display technologies [48]. Another intriguing detail is that in their study, TNA was first linked to its complementary DNA and amplified as a double strand so that reverse transcriptase was no longer necessary.

XNA aptamers have exceptional affinity and specificity compared to traditional DNA or RNA aptamers. Gordon et al. used click chemistry to synthesize a mannose-modified nucleic acid aptamer that showed unusual specificity for Concanavalin A and also exhibited significant biological activity, being the most potent inhibitor of Concanavalin A-mediated hemagglutination reported to date [49], highlighting the potential application of XNA aptamers. However, due to the high cost of synthesizing XNA aptamers using the current technologies, the relevant studies are still relatively scarce. Ideally, XNA aptamers would specifically bind to their targets in vivo without affecting other normal tissues, but due to the wide range of XNA aptamers that can be modified, there is no mature theory, and their pharmacological properties have yet to be investigated.

## 4. Aptamer Screening Independent of Intermediate Nucleic Acid Amplification

**Figure 3:**
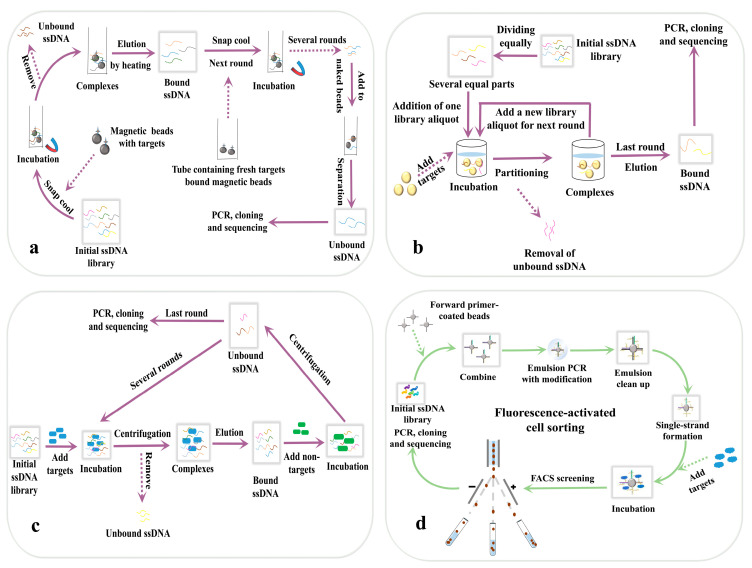
The schematic diagrams of aptamer screening methods independent of intermediate nucleic acid amplification. (**a**): The principle of magnetic bead-assisted screening. The target is immobilized on magnetic beads and incubated with the addition of the nucleic acid library, then the bound and unbound aptamers are separated with a magnet. The magnetic beads are collected using a magnetic rack, and the sequences bound to the target are eluted and added to the next microcentrifuge tube containing fresh target-coupled magnetic beads for a new round of screening. The aptamers from the final round of screening are incubated with naked magnetic beads to exclude sequences that are specifically bound to the beads [64]; (**b**): The principle of competition-enhanced ligand screening. The random nucleic acid library is divided into several aliquots and then one of the aliquots is taken first and added to the target. After incubation, the unbound sequences are washed and removed. A new aliquot of the library is then added to the target–DNA complex and the newly added sequence competes with the bound sequences for the target [65]; (**c**): The principle of centrifugal distribution method. After incubation of the random nucleic acid library with the target, a high-speed centrifugal filter is used to separate the complex from the free oligonucleotide. Negative selection and positive selection are repeated alternately to obtain specific sequences [66]; (**d**): The principle of fluorescence-activated cell sorting. Cells are specifically labeled with fluorescent dyes and then the fluorescent signals are excited by the laser of a flow cytometer to classify and sort the cells according to different fluorescent signal intensities. This technique allows for large-scale screening [49].

Achieving aptamer selection without the need of nucleic acids amplification not only reduces the time and resources required for the entire process, but also offers advantages over traditional techniques for screening RNA and XNA aptamers. These non-SELEX methods provide a perfect screening efficiency and accuracy, with reliable results even in one round of selection. Some high-efficiency partitioning methods are not preferred due to difficulties in connecting with the amplification process in the earlier stages of aptamer research, but are more suited for non-SELEX methods. In this section, we still classify these methods depending on the status of the aptamer. The principles of these methods are displayed in Figure 3, whereas the details of the related works are presented in Table 2.

### 4.1. Non-SELEX Methods in Immobilized Format

#### 4.1.1. Magnetic Bead-Assisted Screening

The classical magnetic beads-SELEX (MBs-SELEX) combines SELEX technology with magnetic bead separation, using magnetic beads as a medium for the immobilization of target molecules and exploiting the magnetic properties of the beads to achieve the rapid and efficient separation and purification of nucleic acid molecules. This method facilitates the separation process, but is not favorable for amplification, which can be avoided in the non-SELEX technique. Gordon et al. immobilized a library of modification-based aptamers synthesized by click chemistry on magnetic beads and used the magnetic field as a driving force to complete the whole screening process [49]. In 2022, the magnetic bead-based non-SELEX method was reported for the β-tyrosinol peptide-7-specific aptamer screening [64]. In total, five rounds of screening were performed and then naked beads were introduced as a negative selection to improve specificity. In addition, magnetic beads are often present as a fixation medium in FACS.

#### 4.1.2. Competition Selection

The Systemic Evolution of Ligands by COmpetitive Selection (SELCOS) is a method of screening aptamers by the competitive selection of targets, which is particularly suitable for screening aptamers that can discriminate between highly similar targets [67]. It is a modification of SELEX based on a competitive strategy. Traditional competitive SELEX introduces multiple targets during each round of screening, incubating libraries, target molecules, and other competitors together to improve screening efficiency by increasing the selection pressure. SELCOS has a similar strategy, but without the repetitive binding, washing, isolation, and amplification processes of traditional competitive SELEX. Kushwaha et al. used this method to successfully obtain DNA aptamers against influenza virus subtypes and detected them rapidly and accurately using an electrochemical sensor [68].

Another method, called competition-enhanced ligand screening (CompELS), also relies on competition rather than evolution to screen aptamers, and is suitable for the rapid screening of large nucleic acid libraries [65,69]. Just as its name implies, the random sequence library is introduced repeatedly between cycles, and the sequences that remain bound to the target survive as candidate sequences. After preparing a random nucleic acid library, it is first divided into multiple aliquots, and one ssDNA library aliquot is added to the target. After a period of incubation, the unbound sequences are washed and removed. Then, a new ssDNA library aliquot is added to the target–DNA complexes, where the newly added sequences compete with the already bound sequences for the target. After several rounds of CompELS, the candidate aptamers are obtained by eluting and amplifying the sequences bound in the last round. All the candidate aptamers undergo a round of competitive binding experiments, and their binding properties are effectively sequenced using next-generation sequencing (NGS) technology to determine the best binding sequence. This competitive screening allows not only to obtain aptamers with higher affinity, but also to obtain many different aptamers at once. This is applicable for certain situations, for example, the development of sandwich-type aptamer sensors based on dual aptamers, where two specific aptamers bound to the target simultaneously greatly improved the sensitivity and specificity of the sensor [83]. Additionally, by rationally designing the structure of different aptamers and selecting different signal detection methods, the simultaneous detection of multiple targets can be achieved. For example, Sun et al. developed a chemiluminescence sensor using dual aptamers to achieve the simultaneous detection of α-fetoprotein and carcinoembryonic antigen in serum samples [84]. At present, there are few reports on competition law, and the information obtained is limited. But its emergence provides a new idea for the development direction of non-SELEX.

### 4.2. Non-SELEX Methods in Non-Immobilized Format

#### 4.2.1. Capillary Electrophoresis in Non-SELEX

The capillary electrophoresis-based non-SELEX technology is roughly the same as CE-SELEX in principle and methodology, except that no amplification is required between the two rounds of screening, and the DNA portion collected in the first round is directly used as the input DNA for the second round, which is incubated with a new aliquot of the target and injected into the capillary to start a new round of cycling, which speeds up the screening process [14,66]. Some experiments have demonstrated that the equilibrium dissociation constant values of aptamers screened by this method are comparable or even better than those obtained by classical CE-SELEX and also avoid repeated amplification, which reflects the value of the development of the non-SELEX technology and injects a strong impetus for the in-depth study of non-SELEX. Qu et al. succeeded in isolating complexes bound to two different proteins in the same capillary channel and competition between the two proteins resulted in screening aptamers with higher specificity and affinity [70]. Aptamers that underwent one round of screening had the same performance as those that were obtained after three rounds of traditional CE-SELEX [71].

#### 4.2.2. Centrifugal Distribution Method

The performance of aptamers obtained by repetitive centrifugation-based separation methods is comparable to those screened by conventional SELEX, but is simpler and faster. Jeong et al. used a non-SELEX method based on a centrifugation-based separation method to rapidly isolate *Escherichia coli*-specific DNA aptamers, which omitted the SELEX evolutionary process, and employed sequential centrifugation-based separation which removes pools of DNA libraries that do not bind to the target [72]. Kim et al. also used this method to screen aptamers for *B. carboniphilus* and verified the selectivity of the aptamers by a colorimetric method [73]. In addition, the non-SELEX method based on centrifugal dispensing can be used for the rapid detection of microorganisms in samples, and bacteria can be recaptured even when sprayed into the air [74]. The centrifugal distribution method, although simple and reproducible, is currently predominantly utilized in the development of specific aptamers for bacterial cells, with few applications to other target types in the available research literature. The effectiveness of this screening method is attributed to its dependence on centrifugation and the principle of separating substances according to density and size. Centrifugal distribution is particularly well-suited to large biomolecules or cells. When applied to small molecules, challenges arise in the complete separation of complexes from free sequences during centrifugation, leading to suboptimal screening outcomes in terms of efficiency and specificity. As a result, the centrifugal distribution method is generally unsuitable for screening small-molecule aptamers.

### 4.3. In Silico

Conventional SELEX techniques rely on experimentation, which prevents cost reduction. In silico aptamer screening aims to predict and screen candidate sequences that interact with specific biomolecules through computational simulation and molecular docking techniques, which is an efficient and cost-effective method compared to conventional SELEX screening. Specifically, in the aptamer screening process, computer simulations are first used to construct a structural model of the biomolecule and identify key information such as the active site. Then, the secondary and tertiary structures of the sequences in the library are predicted and molecular docking techniques are used to predict the binding and affinity of these sequences to the target to identify the aptamer that best interacts with the target [75]. Finally, the binding affinity and stability of the aptamers are further evaluated by experimental validation [76]. Mousivand et al. used a method called in silico maturation (ISM) in conjunction with molecular docking techniques to improve the affinity and selectivity of DNA aptamers for aflatoxin B_1_. They first evolved improved aptamer sequences from potential parent sequences by one- and two-point mutations, different crossing over, and selected the sequence with the highest affinity as the final sequence after several rounds of genetic algorithm iteration. The selectivity of the selected aptamer for six different mycotoxins was then evaluated by the molecular docking technique. Finally, an aptamer with good affinity and specificity was obtained, which could be used for the detection and removal of mycotoxins in food [77]. Bell et al. designed and validated high-affinity RNA aptamers targeting an epithelial cellular adhesion molecule (EpCAM). They obtained the structural and kinetic information of the EpCAM by molecular dynamics simulations and predicted the binding mode of the RNA aptamers to the EpCAM. The effects of different mutations on the binding affinity of RNA aptamers to EpCAM were evaluated by free-energy perturbation calculations. In addition, molecular docking was performed to predict the binding sites and binding modes of RNA aptamers to EpCAM. Finally, the binding affinity of the RNA aptamers to EpCAM was confirmed by isothermal titration calorimetry experiments. The results showed that the designed RNA aptamers have high affinity and specificity, and are expected to be used for the targeted therapy of EpCAM [78]. In silico has been developing rapidly in the community and the transition from traditional experiments to virtual screening reflects a shift in the research mindset, with a variety of software and algorithms emerging [85,86]. As such, in recent years, numerous scholars have published many reviews on in silico, which provide a comprehensive and detailed description of the method and interested readers can read them [79,87,88,89,90].

Compared to conventional SELEX, the current application of in silico is not considered to be very widespread, which may be related to the complexity and uncertainty of the modeling, as well as the limited accuracy of the simulation results at the current state-of-the-art. The development of new software and algorithms is an effective way to solve the above problems, but it is a rather difficult process. Nevertheless, we believe that the future of in silico is very promising.

### 4.4. Other Emerging Automated Screening Methods

Automated advances and high-throughput techniques have enriched tools for aptamer selection. The parallel library-based approach is a high-throughput screening strategy that has emerged in recent years. Lozoya-Colinas et al. described a novel method for discovering aptamers [80]. The strategy bypasses the Darwinian evolutionary process and relies on the presence of functionally enhanced side chains to increase the abundance of functional aptamers in an unbiased pool of random sequences. A single round of high-throughput screening identified trimers capable of binding to S1-RBD proteins with binding affinity values comparable to those of aptamers evolved by monoclonal antibodies and traditional in vitro screening protocols, while greatly simplifying the screening process. This approach can accelerate the discovery of oligonucleotide probes by avoiding the traditional multi-round selection process and holds promise for applications in molecular probes and drug design in diagnostic and therapeutic areas. In addition, the emergence of automated screening platforms has significantly improved the screening efficiency and reduced the labor cost. Wu et al. proposed an automated screening platform called a non-natural nucleic acid aptamer array (N2A2), which can rapidly and efficiently screen and characterize millions of base-modified aptamers. These aptamers have superior affinity and specificity relative to their natural DNA or RNA [81]. By using this platform, researchers can discover nucleic acid aptamers with high specificity and strong affinity more quickly and efficiently, providing strong support for research in related fields.

## 5. Conclusions and Future Perspectives

One of the biggest challenges in the current clinical application of DNA aptamers is their poor stability when handling complex biological samples. Susceptibility to nuclease degradation remains a challenge limiting their use [6]. In addition to nuclease degradation, the rapid renal clearance of aptamers is another challenge for clinical applications [91]. RNA aptamers are also plagued by these problems. To overcome these problems, the modification of aptamers to enhance their stability has become one of the hotspots of current research. For example, by synthesizing modified bases, the enzyme resistance and biological half-life of aptamers can be improved, thereby increasing their survival time and efficacy in vivo.

The current challenge in screening RNA and XNA aptamers lies in the amplification process, causing high costs in time and resources. Non-SELEX methods finish screening procedures without amplification and are becoming more and more important to aptamer-based applications. Based on our systematic review, three aspects are crucial to further the development of non-SELEX methods.

First, researchers need to consider how to integrate or adapt high-resolution partitioning techniques into current screening methods so that screening efficiency could be optimized. Directly implementing multiple rounds of screening without the library or without amplification does not require much adjustment to the existing protocols, but the outcome is affected by sample loss during each round. High-resolution partitioning techniques increase the screening efficiency and make it possible to achieve satisfying results within one round of selection. Capillary electrophoresis is the most efficient partitioning technique applied in traditional SELEX, which has nurtured many non-SELEX methods with minor adjustments [66]. However, adaptations with novel partitioning techniques provide a higher throughput. The above-mentioned example N2A2 systems display an unparalleled high throughput and resolution. However, we believe that there are many other techniques that are capable for this mission that have not yet been applied in aptamer screening. For instance, surface plasmon resonance imaging (SPRi) could simultaneously monitor the weight variation of different regions on a gold prism [2]. Even though SPR phenomena are widely used in studying interactions between nucleic acids and other molecules, SPRi is seldom used for aptamer selection. The current limitation is possibly due to the throughput. Although SPRi could screen a few dozen spots on the prism, it is not sufficient to support the screening of the whole library on one chip.

Second, more efforts should be put into the study of the binding mechanism between different types of nucleic acids and targets. Many researchers have studied the mechanism of aptamer–target interactions using various methods such as thermodynamic analysis, nuclear magnetic resonance and molecular simulation [92,93,94,95]. However, a comprehensive and clear explanation has not yet been achieved. Exploring this mechanism may help us discover a more efficient screening method, which can be a potential research direction for aptamer screening in the future.

Last but not least, more strategies for virtual screening and the development of new algorithms for in silico should be explored. The current research has shown that virtual screening techniques can save a lot of time and materials, but unfortunately, existing software is difficult to accurately simulate three-dimensional oligonucleotide structures or non-canonical base pairing and has not been able to achieve applications in non-natural nucleic acids [82]. Breaking through this difficulty requires the development of new software and algorithms.

## Figures and Tables

**Table 1:** Performance and details of aptamers screened with SELEX methods in the current literatures.

Method	Target Type	Target	Type of Aptamer	K_d_	Reference
Capture-SELEX	Small molecule	Sterigmatocystin	DNA	25.3 nM	[25]
Capture-SELEX	Small molecule	Salicylic acid	DNA	26.7 ± 5.8 μM	[26]
Immobilized metal affinity chromatography-SELEX	Polypeptide	A histidine-tagged 29 amino acid peptide	DNA	-	[27]
Affinity chromatography-SELEX	Antibody	Lucentis	DNA	23–35 nM	[28]
Atomic force microscope-SELEX	Protein	NCYM	DNA	53.9–299 nM	[29]
Capillary Electrophoresis-SELEX	Protein	Shellfish Allergen Tropomyosin	DNA	0.95 nM	[18]
Microbead-assisted capillary electrophoresis-SELEX	Small molecule	Methotrexate	DNA	0.57 μM	[30]
Three-step evolutionary enhanced capillary electrophoresis-SELEX	Protein	Natural killer cells exosome-specific proteins	DNA	27.6 nM	[31]
Ideal-Filter Capillary Electrophoresis	Protein	MutS protein	DNA	40 nM	[32]
Fluorescence-activated cell sorting	Protein	RNase B; Fetuin	Modified DNA	29.5 ± 2.7 µM; 6.2 ± 0.2 µM	[33]
Two-step SELEX	Cell	The cell membranes of clinical cancer tissues	DNA	41.6 ± 8.7 nM	[34]
3D Cell-SELEX	Cell	SKBR3 cells	DNA	81.4 nM	[35]
Graphene Oxide-SELEX	Small molecule	Cyclosporine A	DNA	41.21 ng/mL	[20]
Graphene Oxide-SELEX	Small molecule	Tramadol hydrochloride	DNA	178.4 nM	[36]
Tripartite-hybrid SELEX	Protein	LipL32 protein of Leptospira	RNA	350 ± 47.45 nM	[37]
SELEX	Protein	DasR protein	RNA	406 ± 15 nM	[38]
Cell internalization SELEX	Cell	B-ALL cells	RNA	-	[39]
Microfluidic screening	Small molecule	Theophylline	RNA	1.7 ± 0.3 μM	[40]
Microfluidic-assisted in vitro compartmentalization	Aptamer	The light-up RNA aptamer SRB-2	RNA	-	[41]
Capillary Electrophoresis-SELEX	Protein	PD-L1 protein	TNA	400 nM	[42]
Cell internalization SELEX	Cell	T24 cancer cells	Modified RNA	-	[43]
Affinity chromatography-SELEX	Enzyme	HIV reverse transcriptase	TNA	1–15 nM	[44]
Hybrid-Type SELEX	Protein	Human TROP2	Artificial nucleic acid	50 ± 6.9 nM	[45]
Split−Combine Click-SELEX	Cytokine	CXCL9	Clickmer	12 ± 2 nM; 92 ± 14 nM	[46]
Highly Parallelized Screening	Protein	The full S1 glycoprotein of SARS-CoV-2	TNA	1–300 nM	[47]
Display strategy based on TNA	Enzyme	HIV reverse transcriptase	TNA	~0.4–4.0 nM	[48]
Click-particle display	Small molecule; Protein	Epinephrine; Concanavalin A	Modified DNA	~1.1 µM; 3.2 ± 0.2 nM	[49]

**Table 2:** Performance and details of aptamers screened with non-SELEX methods in current literature.

Method	Target Type	Target	Type of Aptamer	Kd	Reference
Non-SELEX assisted by magnetic beads	Polypeptide	β-casomorphin-7	DNA	28.93 ± 0.783 nM	[64]
Competition-based aptamer selection strategy	Protein	CYP24A1	DNA	-	[67]
Systematic evolution of ligands by competitive selection	Protein	Influenza virus protein H1N1	DNA	82 pM	[68]
Competition-enhanced ligand screening	Metal	PlanarAu	DNA	0.56 nM	[65]
Competition-enhanced ligand screening	Nanomaterial	Gold nanorod	DNA	-	[69]
Non-SELEX based on the capillary electrophoresis	Protein	Tau protein	DNA	13 ± 3; 116 ± 6; 84 ± 6; 49 ± 4 nM	[66]
One-round pressure controllable selection	Protein	Human holo-transferrin; Platelet-derived growth factor-BB	DNA	0.050 ± 0.015; 0.081 ± 0.018 µM	[70]
One-round pressure controllable selection	Protein	8-Oxoguanine DNA glycosylase	DNA	1.71~2.64 µM	[71]
Centrifugation-based partitioning method	Bacterium	*Escherichia coli*	DNA	101.76 nM	[72]
Centrifugation-based partitioning method	Cell	*Escherichia coli* cell	DNA	3.9 ± 0.6; 8.0 ± 0.9; 10.1 ± 0.7 nM	[73]
Centrifugation-based partitioning method	Bacterium	*Citrobacter braakii*	DNA	16.42 ± 2.30 nM	[74]
In silico screening strategy	Protein	Epithelial cell adhesion molecule	RNA	21.8 ± 3.1; 96.9 ± 25.65 nM	[75]
In silico screening strategy	Protein	*Streptoccocus agalactiae* surface protein	RNA	-	[76]
In silico maturation strategy	Toxin	Alatoxin B_1_	DNA	0.004–8.7 nM	[77]
In silico screening strategy	Protein	Epithelial cell adhesion molecule	RNA	39.89 ± 3.37 nM	[78]
In silico screening strategy	Protein	Neuron-specific enolase	DNA	2.76 ± 1.14 nM	[79]
Parallelized library screening	Protein	The S1 protein from SARS-CoV-2	XNA	0.8–3.7 nM	[80]
Non-natural aptamer array (N2A2) system	Protein	Vascular endothelial growth factor; Fetuin; Insulin	Modified DNA	2.8 nM ± 0.63; 3 µM; 4.8 µM	[81]
Parallel screening-based strategy	-	ATP	DNA	12–157 μM	[82]

## Data Availability

Data availability is not applicable to this article as no new data were created or analyzed in this study.

## Abbreviations

SELEX: Systematic Evolution of Ligands by Exponential Enrichment; XNA, Xeno nucleic acids; FAM, fluorescein Amidite; ssDNA, single strand DNA; IMAC, immobilized metal affinity chromatography; IAC, immunoaffinity columns; AAC, aptamer affinity columns; AFM, atomic force microscope; CE, capillary electrophoresis; LoD, Limit of Detection; FACS, fluorescence activated cell sorting; 2D, two-dimensional; 3D, three-dimensional; GO, graphene oxide; μIVC, microfluidic-assisted in vitro compartmentalization; ANN, artificial neural network; TNA, α-L-threofuranosyl nucleic acid; MBs-SELEX, magnetic beads-SELEX; SELCOS, Systemic Evolution of Ligands by COmpetitive Selection; CompELS, competition-enhanced ligand screening; NGS, next-generation sequencing; ISM, in silico maturation; EpCAM, epithelial cellular adhesion molecule; N2A2, non-natural nucleic acid aptamer array; SPRi, surface plasmon resonance imaging.
